# Capturing patient-reported sleep disturbance in atopic dermatitis clinical trials

**DOI:** 10.1186/s41687-024-00751-7

**Published:** 2024-07-15

**Authors:** Carla Dias-Barbosa, Jonathan I. Silverberg, Sonja Ständer, Danielle Rodriguez, Fatoumata Fofana, Dina Filipenko, Liliana Ulianov, Christophe Piketty, Jorge Puelles

**Affiliations:** 1 Evidera, Ivry-sur-Seine, France; 2 George Washington University School of Medicine and Health Sciences, Washington, D.C. USA; 3 Department of Dermatology and Center for Chronic Pruritus, University Hospital Münster, Münster, Germany; 4 Evidera, Seattle, WA USA; 5 Evidera, Bennekom, The Netherlands; 6 Evidera, London, UK; 7 Galderma, Zug, Switzerland

**Keywords:** Content validity, Dermatitis, Atopic, Patient reported outcome measures, Psychometrics, Sleep Wake disorders

## Abstract

**Background:**

Patient-focused approaches to capturing day-to-day variability in sleep disturbance are needed to properly evaluate the sleep benefits of new treatments. Such approaches rely on patient-reported outcome (PRO) measures validated in the target patient population.

**Methods:**

Using atopic dermatitis (AD) as an example of a disease in which sleep is commonly disturbed, we developed a strategy for measuring sleep disturbance in AD trials. In developing this strategy, we conducted a targeted literature review and held concept elicitation interviews with adolescents and adults with AD. We subsequently identified potentially suitable PRO measures and cognitively debriefed them. Finally, we evaluated their psychometric properties using data from phase 2b (NCT03100344) and phase 3 (NCT03985943 and NCT03989349) clinical trials.

**Results:**

The literature review confirmed that sleep disturbance is a key impact of AD but failed to identify validated PRO measures for assessing fluctuations in sleep disturbance. Subsequent concept elicitation interviews confirmed the multidimensional nature of sleep disturbance in AD and supported use of a single-item measure to assess overall sleep disturbance severity, complemented by a diary to capture individual components of sleep disturbance. The single-item sleep disturbance numerical rating scale (SD NRS) and multi-item Subject Sleep Diary (SSD)—an AD-adapted version of the Consensus Sleep Diary—were identified as potentially suitable PRO measures. Cognitive debriefing of the SD NRS and SSD demonstrated their content validity and their understandability to patients. Psychometric analyses based on AD trial data showed that the SD NRS is a well-defined, reliable, and fit-for-purpose measure of sleep disturbance in adults with AD. Furthermore, the SD NRS correlated with many SSD sleep parameters, suggesting that most concepts from the SSD can be covered using the SD NRS.

**Conclusions:**

Using these findings, we developed an approach for measuring sleep disturbance in AD trials. Subject to further research, the same approach could also be applied to future trials of other skin diseases where itch causes sleep disturbance.

**Supplementary Information:**

The online version contains supplementary material available at 10.1186/s41687-024-00751-7.

## Background

In 2022, the U.S. Food and Drug Administration (FDA) published a draft roadmap to help guide the measurement of patient-focused outcomes in clinical trials of new medical products [1]. The roadmap has three steps: (1) understanding the disease; (2) conceptualizing the benefits and risks of treatment; and (3) selecting or developing outcome measures. The first step is to understand how the disease manifests and progresses. This often involves conducting qualitative interviews to seek the perspectives of patients on signs and symptoms, impacts on daily life, and unmet treatment needs. The second step involves determining which concepts (disease experiences) are important to patients and should be targeted by the medical product. These experiences are used to develop outcomes and endpoints for clinical trials. Finally, the third step establishes whether a PRO measure already exists to capture the outcomes. A literature search can be performed to identify existing measures, which are then assessed to determine whether they are properly validated for the proposed use or require further validation prior to being used. If no appropriate PRO measure is identified, then a new one will need to be developed.

Patients with the inflammatory skin disease atopic dermatitis (AD) often experience intense pruritus (itch) [2]. This, together with skin pain, can cause sleep disturbance, which in turn leads to daytime sleepiness and functional impairment [3–5]. Although sleep disturbance is an important contributor to the disease burden of AD, it has not been generally prioritized in the clinical trial endpoint hierarchy. Objective measures of sleep such as actigraphy and polysomnography have been included in some AD trials [6, 7] but lack a direct patient perspective for interpreting a meaningful treatment effect. Nor can they capture the patient’s perspective or distinguish sleep disturbance due to AD symptoms from sleep disturbance due to other causes.

Patient-focused strategies for capturing day-to-day variability in sleep disturbance are needed to properly evaluate the sleep benefits of new treatments. Various PROs are available for assessing sleep quality [8, 9]. However, to be useful in assessing treatment effects on sleep, they need to be validated in the target patient population. Focusing on AD as a disease in which sleep is commonly disturbed, we used the FDA roadmap to develop a patient-focused strategy for measuring sleep disturbance in clinical trials.

## Methods

**Fig. 1:**
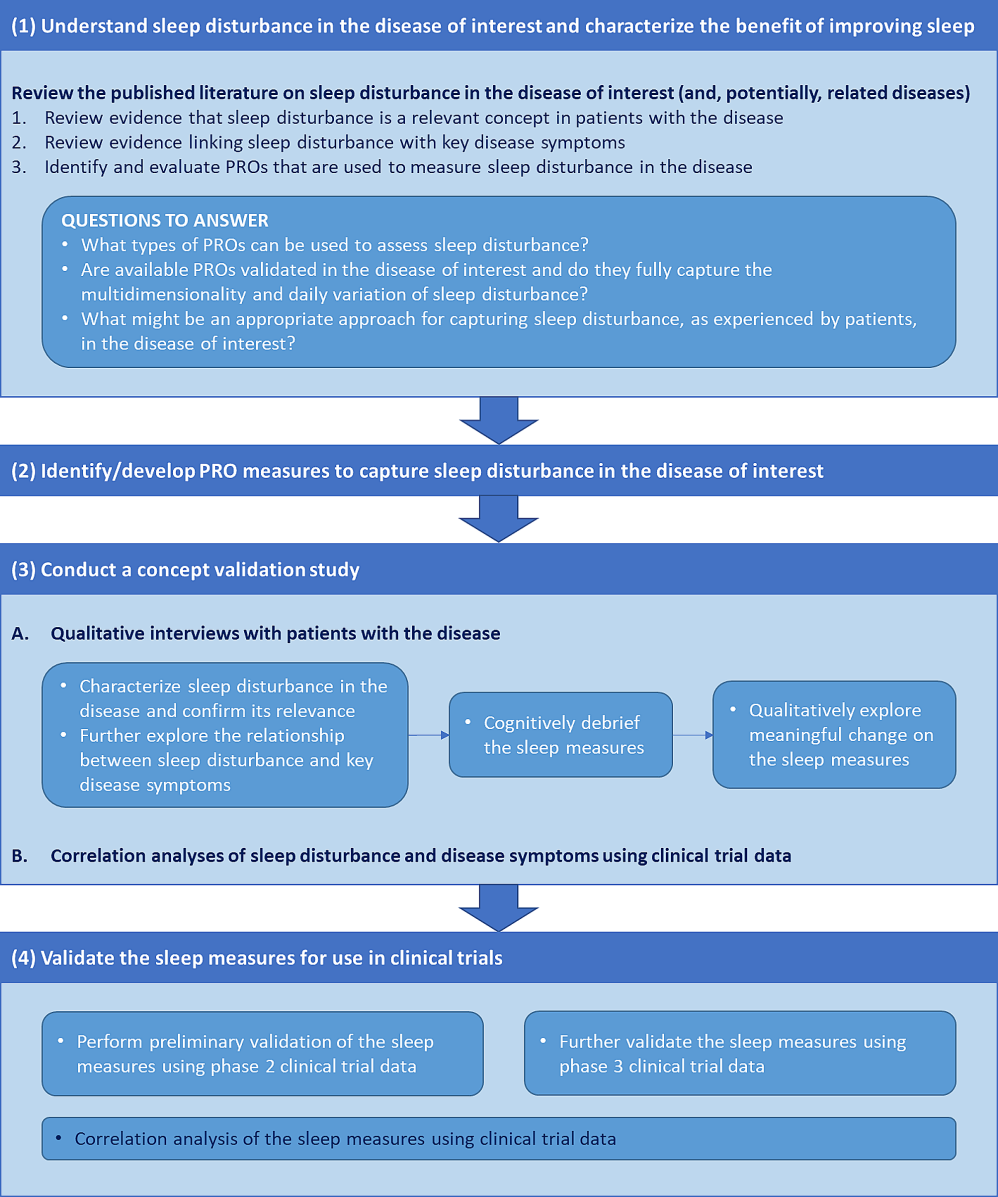
Four-step strategy for measuring sleep disturbance in clinical trials. *PRO* patient-reported outcome

In developing the strategy for measuring sleep disturbance in AD, we adopted a four-step approach (Fig. 1). Components of the approach involving human subjects received the necessary ethics committee approval and were conducted in accordance with the Declaration of Helsinki.

### Literature review

First, a literature review with pre-defined search terms was conducted in Medline and Embase to retrieve and evaluate evidence supporting sleep disturbance as a relevant concept in patients with AD and to identify PRO measures used to measure sleep disturbance in AD and other skin diseases. Included literature comprised full-text articles and abstracts reporting research in subjects aged ≥ 13 years and published in English between January 1, 2014, and August 1, 2019. The literature was assessed using pre-defined criteria for inclusion of phase 2 and 3 clinical trials, observational and PRO instrument development studies, targeted literature reviews, qualitative meta-syntheses, meta-analyses, and systematic literature reviews. Studies in non-skin diseases or non-human populations, in vitro studies, basic science research, and non-English articles were excluded. The literature was reviewed to assess the link between sleep disturbance and AD; to evaluate the relationship between itch and sleep disturbance in AD and other pruritic skin diseases; and to identify and evaluate existing PRO measures that have been used to assess sleep disturbance in skin diseases.

### Identification of potentially suitable PRO measures

Symptoms such as itch or pain can often be assessed using single-item instruments. By contrast, multidimensional disease impacts such as sleep disturbance may require a holistic approach combining a single-item measure to ease interpretation of a clinically meaningful treatment effect and a multi-item instrument to capture the multidimensionality of the concept. For example, a single-item instrument can capture overall sleep disturbance during the previous night, whereas a multi-item diary can capture additional aspects of a patient’s sleep, such as how symptoms (e.g., itch) affect their ability to fall asleep and get back to sleep, the duration and quality of their sleep, the duration and frequency of nighttime awakenings, how refreshed and rested they feel in the morning, and daytime sleepiness. Based on these considerations, the single-item sleep disturbance numerical rating scale (SD NRS) and the Subject Sleep Diary (SSD)—a 15-item, AD-adapted version of the Consensus Sleep Diary—were selected for validity testing.

### Establishing the content validity of the selected PRO measures in the target population

As a first step in determining whether the selected PRO measures might be suitable for measuring sleep disturbance in AD patients, adolescents (age 12–17 years) and adults (age ≥ 18 years) resident in the US took part in concept elicitation interviews [10]. The interview participants had a clinical diagnosis of AD that was moderate-to-severe (Eczema Area and Severity Index [EASI] score ≥ 12 within 2 weeks of enrolment) [11, 12]. Interviews were conducted by telephone using a semi-structured interview guide. Participants were asked open-ended questions about their experiences of AD and sleep disturbance. During the same interviews, the SD NRS and SSD were cognitively debriefed [10]. Further, participants were probed on the meaningfulness of a 1-, 2-, or 3-point change on the SD NRS. Not all participants provided a response to some questions. Percentages were calculated with the number of respondents for each question as the denominator.

Based on feedback from these initial interviews, the SSD was revised. A convenience sample of participants from the first round of interviews then completed the revised version of the SSD and were re-interviewed about it. Finally, themes, concepts, and descriptions of sleep disturbance from the concept elicitation interviews were mapped to items/sleep metrics included in or derived from the final version of the SSD.

### Correlation analysis of sleep disturbance

The relationship between sleep disturbance and itch in AD was further examined by multivariate linear regression using data from a 24-week phase 2b placebo-controlled trial of the anti-interleukin-31 receptor A monoclonal antibody nemolizumab in adults (≥ 18 years) with moderate-to-severe AD based on an EASI score of ≥ 12 and an Investigator Global Assessment (IGA) score of ≥ 3 (NCT03100344) [13, 14]. Sleep disturbance was assessed using the SD NRS, and itch was assessed with two other single-item measures: the peak pruritus numerical rating scale (PP NRS) [15] and Average Pruritus Numerical Rating Scale (AP NRS) [16]. Participants completed the sleep and itch measures daily throughout the trial. Weekly average scores were calculated for the SD NRS, PP NRS, and AP NRS and were used in the analysis.

### Psychometric evaluation of the single-item PRO measure

#### Initial validation

Data from the same phase 2b trial of nemolizumab were additionally used to assess the psychometric properties of the SD NRS [17]. Test-retest reliability, construct validity, known-groups validity, and responsiveness were all analyzed. Other PROs used in these assessments were the Pruritus Categorical Score (PCS) [18], PP NRS, AP NRS, 5-D Itch Scale [19], EQ-5D-3 L [20], Dermatology Life Quality Index (DLQI) [21], and Hospital Anxiety and Depression Scale (HADS) [22]. Clinician-reported outcome measures included the SCORing Atopic Dermatitis (SCORAD) sleep loss visual analog scale (VAS) [23], EASI, and IGA [13].

Following draft FDA guidance [24], a meaningful within-patient score difference representing meaningful improvement on the SD NRS for adults with AD was estimated by anchor-based methods using the phase 2b trial data [14, 17]. Supportive evidence came from distribution-based estimates [17], as well as from qualitative findings for meaningful change (see above) [10]. Results of the anchor- and distribution-based, and qualitative analyses were triangulated to obtain a range of thresholds for meaningful score differences on the SD NRS.

#### Final psychometric validation

Data from two 16-week phase 3 placebo-controlled trials of nemolizumab in adolescents and adults (age ≥ 12 years) with moderate-to-severe AD—ARCADIA 1 (NCT03985943) and ARCADIA 2 (NCT03989349)—were used to confirm the psychometric properties of the SD NRS. The phase 3 psychometric analyses used similar methodology as in the initial phase 2b validation. Test-retest reliability, construct validity, known-groups validity, and responsiveness were analyzed using the same PROs and clinician-reported outcome measures as were used in the initial psychometric validation with phase 2b data, as well as the Patient Global Impression of Severity-Pruritis, Patient Global Impression of Change-Pruritis, Patient Global Impression of Severity-Sleep Disturbance (PGIS-SD), Patient Global Impression of Change-Sleep Disturbance (PGIC-SD), Patient Global Assessment of Disease (PGAD), Patient-Oriented Eczema Measure (POEM) [25], Children’s Dermatology Life Quality Index (cDLQI) [26], Patient-Reported Outcomes Measurement Information System (PROMIS) Itch Questionnaire [27], and Work Productivity and Activity Impairment: Atopic Dermatitis [28]. Also, the range of thresholds for meaningful score differences obtained in the initial validation using phase 2b trial data was further evaluated using phase 3 trial data.

### Correlation analysis of the SD NRS and SSD

Data from the phase 3 trials were also used to evaluate associations between scores on the single-item measure and sleep parameters derived from the multidimensional instrument. Pearson correlation coefficients were calculated for baseline and for changes from baseline to weeks 8 and 16. Correlations were graded as weak (r<|0.30|), moderate (|0.30|≤ r<|0.50|), or strong (r≥|0.50|) [29].

## Results

### Understand sleep disturbance in the disease of interest and characterize the benefit of improving sleep

The literature review of article published been January 1, 2014, and August 1, 2019, identified 41 studies, of which 12 focused on AD and 13 focused on psoriatic arthritis or psoriasis. The remaining 16 studies addressed patients with eczema, urticaria, pruritus, prurigo nodularis, or systemic lupus erythematosus. Twenty-nine studies were observational (24 were cross-sectional; five were longitudinal). Of the remaining 12 studies, five involved PRO development or validation, one was a literature review, and six were phase 2 or 3 randomized controlled trials. No qualitative studies were identified. Nine of the identified studies described the association of sleep disturbance with AD [5, 30–37]. Evaluation of these studies confirmed the link between AD and sleep disturbance. In a study based on the U.S. National Health and Wellness Survey, patients with AD had a higher prevalence of sleep disorders than people without AD [31]. In another survey of 287 adults with AD, only 22% of participants reported good or very good sleep quality in the previous week [5]. Moreover, several interventional studies demonstrated that successful treatment of AD led to a reduction in patient-reported sleep disturbance [30, 35, 37], suggesting that sleep is a relevant treatment outcome in AD trials.

More broadly, sleep disturbance was found to be a feature of pruritic skin diseases. One study describing the development of a conceptual model for chronic itch in adults concluded that sleep disturbance is one of the most important aspects of the disease burden of itch, and that itch and scratching cause sleep disturbance [36]. A further survey found that itch was a predictor of abnormal sleep patterns among patients with pruritus, AD, and two other pruritic skin diseases (psoriasis and prurigo nodularis) [38].

### Identify/develop one or more PRO measures to capture sleep disturbance in the disease of interest

The literature review identified 34 PROs that had been used to assess sleep problems or sleep disorders among patients with skin diseases. The PROs that were most frequently used to assess AD-related sleep disturbance were the single-item SCORAD sleep loss VAS [23] and five multi-item outcome measures: the POEM [25], Pittsburgh Sleep Quality Index (PSQI) [39], Medical Outcomes Study (MOS)-Sleep [40], PROMIS sleep disturbance [41], and PROMIS sleep-related impairment [41]. Collectively, the available literature demonstrated that sleep disturbance can be assessed using self-report measures. However, none of the identified PRO measures was specifically designed or validated for assessing sleep disturbance in patients with AD. Instruments such as the MOS-Sleep, PROMIS sleep disturbance, and PSQI are relevant in terms of coverage but fail to accurately capture day-to-day fluctuations in sleep disturbance. Moreover, patients using these instruments may have difficulties averaging their responses using a recall period of 1 week or more.

The sleep disturbance numerical rating scale (SD NRS) is a single-item self-report measure for scoring the severity of sleep loss related to AD on a scale of 0 (no sleep loss) to 10 (I did not sleep at all) [10]. It was newly developed to assess the overall severity of sleep disturbance related to signs/symptoms of AD and was designed to be completed daily.

The Consensus Sleep Diary^©^ is a standardized multidimensional instrument for monitoring subjective sleep in sleep disorders such as insomnia [42]. The SSD (also known as the CSD-AD^©^) is a version of the Consensus Sleep Diary intended to be used in AD trials [10]. The original Consensus Sleep Diary was modified based on input from clinical experts and results of qualitative concept elicitation and cognitive debriefing interview studies with patients with AD. This allowed the SSD to capture daily sleep patterns, how AD-related symptoms (e.g., itch) affect sleep, and daytime sleepiness. Additional file 1 (Fig. S1) provides an overview of the development and preliminary validation of the SSD. It was anticipated that the SSD could complement the SD NRS by capturing the different components of sleep disturbance (e.g., difficulty falling asleep, nighttime awakenings, difficulty getting back to sleep) individually.

Like the original Consensus Sleep Diary, the SSD captures sleep onset latency, wakefulness after sleep onset (WASO), terminal WASO, total awake time, time in bed, total sleep time, and sleep efficiency. It additionally captures two sleep parameters and one domain specific to AD: duration of WASO related to AD, a sleep parameter captured using the item “*In total, how long did the awakenings related to the symptoms of atopic dermatitis (for example itching, burning) last?*”; number of times of WASO not related to AD, a sleep parameter captured using the item “*How many times did you wake up due to the symptoms of atopic dermatitis (for example itching, burning), not counting the final time you woke up for the day?*”; and sleep quality/refresh, a domain derived by summing the scores for two items: “*How would you rate the quality of your sleep?*” and “*How rested or refreshed did you feel when you woke up for the day?*”

### Conduct a content validation study

#### Concept elicitation interviews

Ten adolescents and 20 adults took part in concept elicitation interviews, which are described in detail elsewhere [10]. Briefly, saturation of concepts was achieved after interviews with four adults and two adolescents. Overall, the most frequently reported sleep problems were nighttime awakenings (87%), trouble falling asleep (73%), feeling unrested (53%), daytime fatigue or sleepiness (53%), and a feeling of not getting enough sleep (33%). Waking early in the morning was reported by 40% of adolescents but only 5% of adults. Notably, 70% of adults and 30% of adolescents experienced sleep disturbance every night, although sleep disturbance varied from day to day for most participants. A third of adults (32%) reported an impact on work, and two thirds of adolescents (67%) reported an impact on school.

Thirty-seven percent of adults and 43% of adolescents attributed their sleep disturbance solely to AD-associated itch [10]. The other participants attributed it to a combination of AD-related symptoms (itch, burning sensation, inflammation, or pain) and non-AD factors (such as needing to go to the bathroom or drink water).

In summary, the concept elicitation interviews confirmed that sleep disturbance in AD is a multidimensional concept, with components including reduced duration of sleep, difficulty falling asleep, nighttime awakenings, early morning awakening, not feeling rested in the morning, and daytime fatigue or sleepiness. All these sleep disturbance-reported concepts are captured by the SSD. Patients also reported on their overall experience of sleep disturbance in general, and this is captured by the single-item SD NRS.

#### Cognitive debriefing of the SD NRS and SSD

Cognitive debriefing of the SD NRS and SSD is described in detail elsewhere [10]. Briefly, all participants understood the SD NRS question as intended. All participants except one adolescent found the SD NRS easy or very easy to understand, and all participants except one adult understood the anchors (descriptors defining the ends of the scale).

The original version of the SSD used in the cognitive debriefing consisted of nine morning items assessing different aspects of sleep during the previous night and two evening items assessing daytime naps and dozing. All adolescents and 90% of adults had a favorable opinion of the SSD [10]. Importantly, all participants were able to remember the number of times they woke up in the night due to itch, and most adults (80%) and all adolescents were able to distinguish nighttime awakenings due to itch from those due to other causes. Moreover, most adults (95%) and all adolescents were able to recall the overall duration of their itch-related nighttime awakenings. This indicated that the SSD was able to specifically capture sleep disturbance related to AD. For both adults and adolescents, the mean number of nighttime awakenings due to itch was higher than the number of nighttime awakenings due to other causes.

Based on the interviews, some SSD items were reworded, and the evening items combining daytime naps and dozing were divided (Additional file 1: Fig. S1). An item on use of sleep medicines was removed, and a new item asking participants what time they got out of bed was added. For nighttime awakenings, existing items on the frequency and duration of itch-related and unspecified awakenings were respectively replaced with items on awakenings related to AD symptoms (with itching and burning given as examples)—to cover all AD symptoms that can cause sleep disturbance—and non-AD-related awakenings. The revised version of the SSD included 11 morning items to be completed once daily in the morning (within one hour of getting out of bed, if possible) and four evening items to be completed once daily in the evening.

Overall, most of the 10 participants who were re-interviewed about the revised version of the SSD (six adults, mean age 30 years; four adolescents, mean age 14 years) understood the items as intended. No further changes were made to the SSD as a result of the second round of interviews. Concept mapping indicated that the SSD covered all concepts of importance to patients (Additional file 2: Tables S1 and S2).

#### Interview participants’ perspectives on meaningful change on the SD NRS

When probed on the meaningfulness of 1-, 2-, and 3-point changes, all participants endorsed a change of 1 to 3 points as meaningful, with 94% of adults and 90% of adolescents indicating that they would consider a 1- or 2-point change meaningful [10].

#### Correlation analysis of sleep disturbance and itch using phase 2b trial data

At baseline, average weekly SD NRS score was strongly correlated with average weekly AP NRS score (regression coefficient 0.94, *p* < 0.0001). Moreover, the change in weekly average SD NRS score between baseline and week 24 was strongly correlated with the change in average weekly AP NRS score during the same time interval. These findings support the close link between sleep disturbance and itch in AD and indicate that amelioration of itch is likely to be accompanied by sleep improvements.

### Validate the sleep measures for use in clinical trials

#### Initial psychometric validation of the SD NRS using phase 2b trial data

Initial psychometric validation of the SD NRS is detailed elsewhere [17]. Briefly, Test-retest reliability was demonstrated based on SD NRS scores at baseline and week 1 for patients who were stable on the SCORAD sleep loss VAS, PCS, PP NRS, or AP NRS. Moderate correlations [43] of the SD NRS with SCORAD sleep loss VAS and 5-D Itch Scale sleep item scores at baseline (Spearman correlation coefficient 0.58 and 0.52, respectively) indicated convergent validity [17]. Correlations with the PP NRS and AP NRS were strong (Spearman correlation coefficient 0.84 and 0.81, respectively); those with baseline EQ-5D-3 L, HADS, EASI, and IGA scores were weak. Moreover, the SD NRS showed known-groups validity based on 5-D Itch Scale sleep item and DLQI scores at baseline, but not based on EASI or IGA scores [17].

The responsiveness analysis included all potential anchors for which score changes between baseline and week 24 were moderately or strongly correlated with SD NRS score changes (Spearman correlation coefficient ≥ 0.30). For all these anchors—SCORAD sleep loss VAS, PCS, PP NRS, AP, NRS, and DLQI—SD NRS scores decreased (improved) more in patients classified as “improved” on the anchors than in those classified as “not improved” [17].

#### Final psychometric validation of the SD NRS using phase 3 trial data

Psychometric analyses performed with data from the ARCADIA 1 trial confirmed the test-retest reliability (intraclass correlation coefficient 0.97–0.98) and convergent and divergent validity of the SD NRS. Known-groups validity was demonstrated based on the PGIS-SD, PGAD, PP NRS, DLQI, and cDLQI at baseline and week 16. Responsiveness was demonstrated based on all included outcome measures (PGIS-SD, PGIC-SD, SCORAD total score, SCORAD sleep loss VAS, DLQI total score [adults only], cDLQI total score [adolescents only], cDLQI sleep item score [adolescents only], PGAD, PCS, PP NRS, AP NRS, EQ-5D-3 L index score, EQ-5D VAS, HADS anxiety score, HADS depression score, body surface area score, IGA, and EASI). Similar results were obtained using data from ARCADIA 2.

#### Triangulation to define meaningful score difference for the SD NRS

Using score changes from baseline to week 24 from the phase 2b trial, anchor-based meaningful score difference estimates for the SD NRS based on the SCORAD sleep loss VAS, PCS, PP NRS, and DLQI ranged from 5.6 to 6.7 points. Distribution-based estimates calculated using week 24 SD NRS data were 1.58 for standard error of measurement and 0.81 for 0.5 standard deviations. When probed, 93% of participants endorsed a 1- or 2-point change as meaningful. Collectively, results from the quantitative analyses of phase 2b trial data suggested that a 2- to 6-point decrease on the SD NRS was a meaningful improvement [44]. Similar analyses performed using phase 3 trial data suggested that a 1- to 7-point decrease (ARCADIA 1) or 1- to 6-point decrease (ARCADIA 2) was meaningful. For both the preliminary range identified using phase 2b trial data and the ranges obtained using phase 3 trial data, a natural cut-point of a ≥ 4-point change on the SD NRS represented a meaningful improvement.

#### Correlation analysis of the SD NRS and SSD

To further support the context of use of sleep-related measures and to refine our strategy for measuring sleep disturbance, correlation analyses were conducted using phase 3 trial data. At baseline, the SD NRS had weak correlations with terminal wakefulness after sleep onset, time in bed, total sleep time, number of times of daytime sleepiness related to AD, and duration of daytime sleepiness related to AD (Table 1). Correlations with sleep onset latency were weak to moderate and those with WASO, total awake time, sleep efficiency, number of times of WASO related to AD, and WASO related to AD were moderate. SD NRS was strongly correlated with sleep quality/refresh. Results were similar for changes from baseline to weeks 8 and 16.

**Table 1:** Pearson correlations between the SD NRS and SSD-derived sleep parameters in the ARCADIA 1 (NCT03985943) and ARCADIA 2 (NCT03989349) trials

Sleep parameter^a^	Baseline SD NRS score		Change in SD NRS score from baseline to week 8		Change in SD NRS score from baseline to week 16	
	ARCADIA 1	ARCADIA 2	ARCADIA 1	ARCADIA 2	ARCADIA 1	ARCADIA 2
SOL	0.267***	0.338***	0.258***	0.173***	0.248***	0.198***
WASO	0.403***	0.385***	0.445***	0.373***	0.488***	0.276***
TWASO	−0.001	0.056	−0.009	0.051	−0.013	0.056
TWT	0.376***	0.418***	0.390***	0.349***	0.412***	0.320***
TIB	0.007	0.112**	−0.080*	0.013	−0.043	0.006
TST	−0.270***	−0.245***	−0.341***	0.259***	−0.338***	−0.243***
SE	−0.430***	−0.433***	−0.449***	−0.374***	−0.482***	−0.380***
NWASO-AD	0.409***	0.400***	0.487***	0.416***	0.506***	0.378***
WASO-AD	0.460***	0.441***	0.485***	0.426***	0.509***	0.366***
SQR	−0.660***	−0.666***	−0.637***	−0.619***	−0.648***	−0.604***
NDS-AD	–	0.153***	–	0.089*	–	0.097*
NDS-AD nap	0.156***	–	0.083*	–	0.081*	–
NDS-AD doze off	0.111**	–	0.047	–	0.055	–
DS-AD total duration	–	0.115**	–	0.081	–	0.097*
DS-AD total nap duration	0.131***	–	0.062	–	0.105*	–
DS-AD total doze off duration	0.078*	–	0.077*	–	0.103*	–

*AD* (related to) atopic dermatitis, *DS* daytime sleepiness, *NDS* number of times of daytime sleepiness, *NWASO* number of times of WASO, *SE* sleep efficiency, *SOL* sleep onset latency, *SQR* sleep quality/refresh, *TIB* time in bed, *TST* total sleep time, *TWASO* terminal wakefulness after sleep onset, *TWT* total awake time, *WASO* wakefulness after sleep onset

^a^SSD-derived sleep parameters were assessed at the same time points as the SD NRS

*p < 0.05, **p < 0.01, ***p < 0.001

## Discussion

Sleep disturbance is a common problem in pruritic skin diseases. Our qualitative interviews with patients confirmed that, in AD, sleep disturbance is complex, multidimensional, and often multifactorial. While it can result from non-AD causes, it is often related to AD symptoms such as itch. Indeed, sleep disturbance is a key component of a published non-disease-specific conceptual model of chronic itch [36]. Our literature review showed that AD patients had a greater prevalence of sleep disturbance than people without AD. This suggests that sleep disturbance is a key impact of AD, in agreement with a conceptional disease model of AD published just after the period covered by our literature review [45]. Moreover, our correlation analyses using phase 2b data confirmed the close link between itch and sleep disturbance in AD, indicating that amelioration of itch is likely to be accompanied by sleep improvements. Collectively, these observations underline the importance of capturing improvements in sleep as an outcome of AD treatment.

In exploring the appropriateness of using the SD NRS and SSD daily to capture both the multidimensional nature of sleep disturbance and day-to-day fluctuations in sleep patterns in AD, concept elicitation/cognitive debriefing interviews provided evidence of their content validity in adults and adolescents with moderate-to-severe AD. Importantly, the SSD captured the sleep disturbance-related concepts that were most frequently reported in the concept elicitation portion of the interviews. Moreover, to our knowledge, the SSD is the first instrument for specifically assessing nighttime awakenings caused by AD symptoms, as opposed to all-cause awakenings. Results from correlation analyses indicated that the SD NRS correlated moderately or strongly with many of the SSD sleep parameters. While the SSD can holistically capture the patient experience of sleep disturbance in AD and provide supplementary information compared to the SD NRS, the SD NRS is easier for patients to complete, less burdensome to administer and score, and allows easier interpretation of meaningful change in sleep disturbance.

Post hoc analysis of phase 2b and phase 3 trial data further indicated that the SD NRS is a well-defined, reliable, and fit-for-purpose measure of the overall severity of sleep disturbance in the target population. Moreover, in the phase 2b analysis of construct validity, SD NRS scores were most strongly correlated with PP NRS and AP NRS scores, further supporting that sleep disturbance in AD is mainly related to itch.

**Fig. 2:**
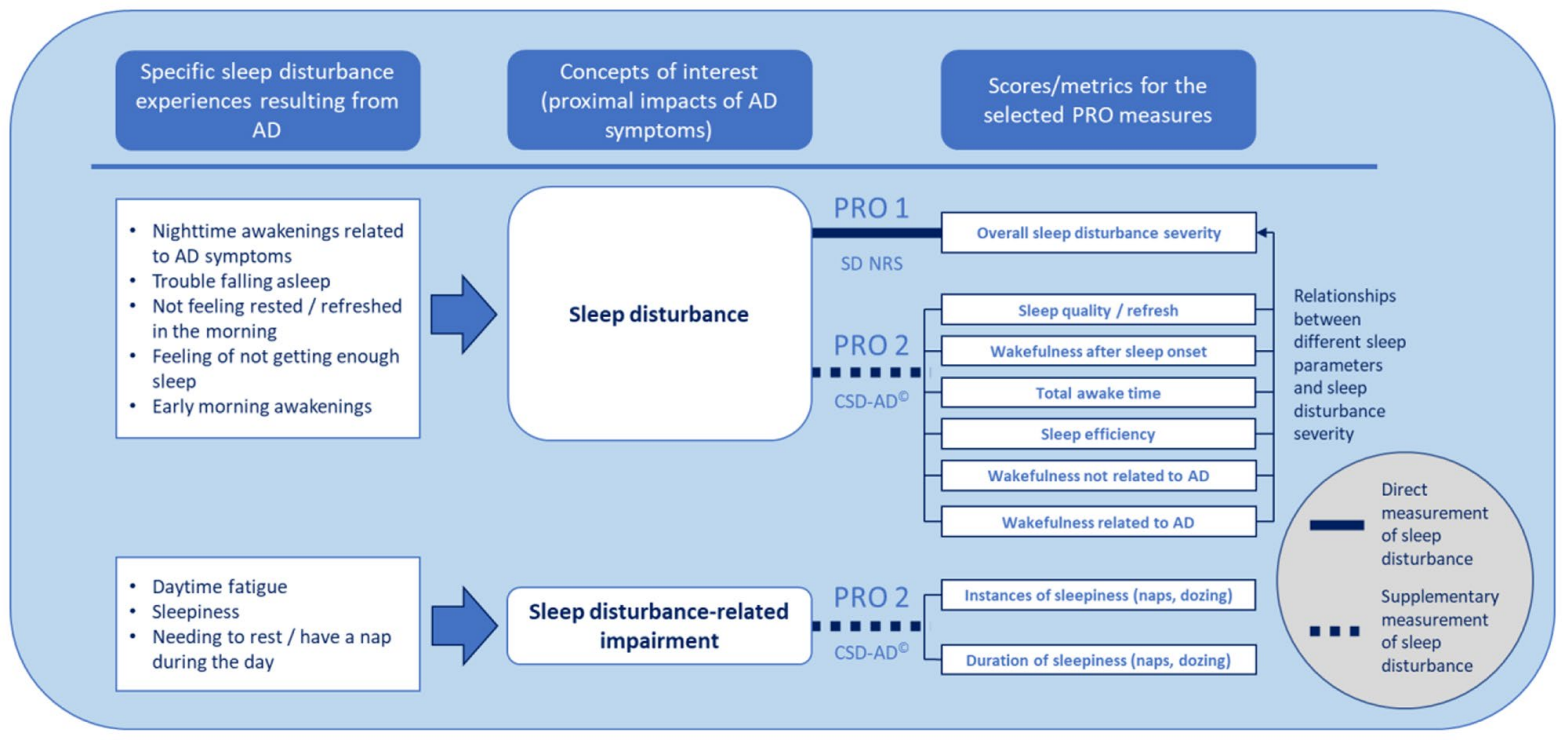
Conceptual framework for measuring sleep disturbance in AD. *AD* atopic dermatitis, *NRS* numerical rating scale, *PRO* patient-reported outcome, *SD NRS* sleep disturbance numerical rating scale, *SSD* Subject Sleep Diary

To our knowledge, there is no standard process for measuring sleep disturbance in AD. To address this, we used patient-focused research findings to develop a new sleep disturbance measurement approach for AD (Fig. 2). The approach includes (1) capturing patient experiences of AD-related sleep disturbance; (2) defining concepts of interest to be assessed; (3) identifying or developing PRO measures for assessing each concept of interest; and (4) deriving domain scores or sleep metrics for the concepts of interest using the PRO measures. Using phase 3 clinical trial data, the relationship between the SD NRS and SSD sleep parameters was evaluated to explore the extent to which a cross-sectional score or score change on the SD NRS correlated with each of the SSD sleep parameters. The sleep disturbance measurement approach was updated to include SSD sleep parameters with moderate to strong correlations with the SD NRS.

Limitations of the present study include not using objective measurement of sleep (e.g., by actigraphy) to support patient report. Moreover, participants in the qualitative research were all resident in the US, although the clinical trial data used in the study were from multinational trials. Finally, the SSD requires additional psychometric validation and score interpretation work.

## Conclusions

The targeted literature review and content validity analysis confirmed the appropriateness of using the SD NRS and SSD to capture the multidimensionality of sleep disturbance and fluctuating sleep patterns in AD. Further, psychometric validation using phase 2b and phase 3 clinical trial data supports the reliability, validity, and responsiveness of the SD NRS in adolescents and adults with AD. The SD NRS correlated with multiple SSD parameters, suggesting that most concepts from the SSD can be covered using the SD NRS. Compared to the SSD, the SD NRS is simpler to complete, easier to administer daily, and allows easier interpretation of meaningful change in sleep disturbance. Subject to further research, the measurement approach we have described here could be applied to other skin diseases where itch is a prominent symptom and a direct cause of sleep disturbance.

### Electronic supplementary material

Below is the link to the electronic supplementary material.

Supplementary Material 1

Supplementary Material 2

Supplementary Material 3

## Data Availability

The datasets used and/or analyzed during the current study are available from the corresponding author on reasonable request.
