# Supplementary material for: Capturing patient-reported sleep disturbance in atopic dermatitis clinical trials
Source: J Patient Rep Outcomes. 2024 Jul 15;8:73. doi: 10.1186/s41687-024-00751-7 (PMC11250737; doi:10.1186/s41687-024-00751-7)
Supplement: Supplementary file 1 — Supplementary Material 1 [file 41687_2024_751_MOESM1_ESM.docx]

Fig. S1. Development and preliminary validation of the SSD


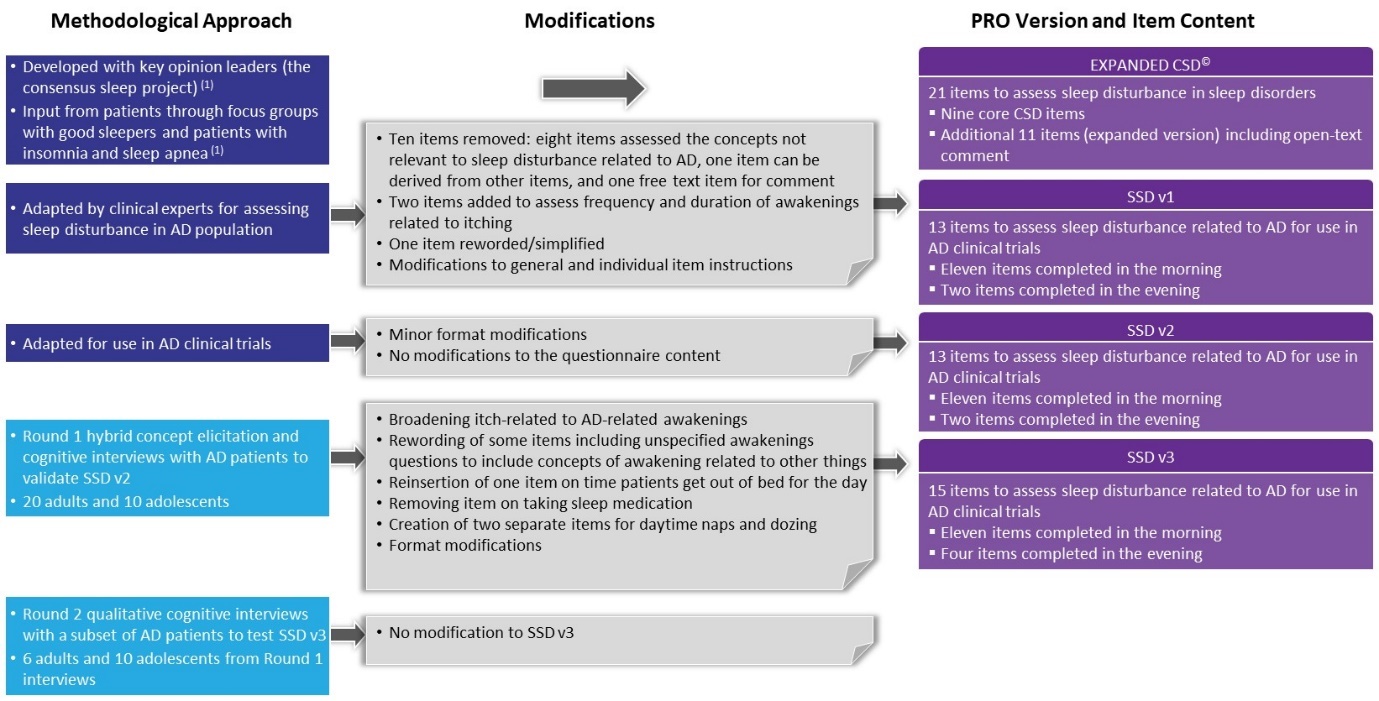


Abbreviations: AD = atopic dermatitis, CSD = Consensus Sleep Diary, SSD = Subject Sleep Diary

^(1)^ Carney CE, Buysse, DJ, Ancoli-Israel S, Edinger JD, Krystal AD, Lichstein KL, Morin CM (2012) The consensus sleep diary: standardizing prospective sleep self-monitoring. Sleep 35:287-302. doi:https://doi.org/10.5665/sleep.1642.
