# Supplementary material for: Capturing patient-reported sleep disturbance in atopic dermatitis clinical trials
Source: J Patient Rep Outcomes. 2024 Jul 15;8:73. doi: 10.1186/s41687-024-00751-7 (PMC11250737; doi:10.1186/s41687-024-00751-7)
Supplement: Supplementary file 2 — Supplementary Material 2 [file 41687_2024_751_MOESM2_ESM.docx]

Table S1. Concept mapping for morning SSD items: sleep disturbance concepts reported in the concept elicitation interviews and the SSD conceptual framework

| **Results from Qualitative Interviews with Patients with AD** | **Adults**  **(N=20)** | **Adolescents**  **(N=10)** | **SSD morning items (11 items)** | | | | | | | | | | |
| --- | --- | --- | --- | --- | --- | --- | --- | --- | --- | --- | --- | --- | --- |
|  |  |  | **Q1m*:**  **Time of getting into bed** | **Q2m*:**  **Time of attempting to fall asleep** | **Q3m:**  **Time it takes to fall asleep** | **Q4m:**  **Number of awakenings related to AD** | **Q5m: Duration of awakenings related to AD** | **Q6m: Number of awakenings related to  other things** | **Q7m: Duration of awakenings related to other things** | **Q8m*:**  **Time of the final awakening** | **Q9m*:**  **Time of getting out of bed** | **Q10m: Perceived sleep quality** | **Q11m: Restfulness after night sleep** |
|  |  |  | **SOL= Q3m; WASO=Q5m+Q7m ; Terminal WASO=Q9m-Q8m ; TWT= SOL+ WASO+ Terminal WASO; TIB: Q9m-Q1m; TST: TIB-TWT; SE: TST/TIB; WASO-AN: Q5m; NWASO-AD: Q4m; SQR: 10+Q11** | | | | | | | | | | |
| **Description of sleep disturbance** |  |  |  |  |  |  |  |  |  |  |  | x |  |
| Nighttime awakenings | 90% | 80% |  |  |  | x | x | x | x |  |  |  |  |
| Trouble falling asleep | 80% | 60% |  |  | x |  |  |  |  |  |  |  |  |
| Feeling unrested | 60% | 40% |  |  |  |  |  |  |  |  |  |  | x |
| Did not get enough sleep | 30% | 40% | x | x | x | x | x | x | x | x | x |  |  |
| Early-morning awakening | 5% | 40% |  |  |  |  |  |  |  | x |  |  |  |
| **Frequency of sleep disturbance** |  |  |  |  |  | x |  | x |  |  |  | x |  |
| Daily | 70% | 30% |  |  |  |  |  |  |  |  |  |  |  |
| Less than daily | 20% | 70% |  |  |  |  |  |  |  |  |  |  |  |
| Did not specify | 10% | 0% |  |  |  |  |  |  |  |  |  |  |  |
| **Duration of sleep per day** |  |  |  |  |  |  | x |  | x |  |  | x |  |
| 3 to 4 hours | 5% | 10% |  |  |  |  |  |  |  |  |  |  |  |
| 4 to 8 hours | 90% | 80% |  |  |  |  |  |  |  |  |  |  |  |
| >8 hours | 5% | 10% |  |  |  |  |  |  |  |  |  |  |  |
| **Sleep variation** |  |  | *To capture daily variations, the SSD should be administered daily* | | | | | | | | | | |
| Depended on flare ups | 32% | 20% |  |  |  |  |  |  |  |  |  |  |  |
| Depended on medication use | 16% | 20% |  |  |  |  |  |  |  |  |  |  |  |
| Depended on weather | 5% | 0% |  |  |  |  |  |  |  |  |  |  |  |
| Varied from day to day but did not provide reason | 26% | 60% |  |  |  |  |  |  |  |  |  |  |  |
| Depended on severity or extent of itching | 5% | 0% |  |  |  |  |  |  |  |  |  |  |  |
| Sleep did not vary | 16% | 0% |  |  |  |  |  |  |  |  |  |  |  |
| **Awakenings per night** |  |  |  |  |  | x |  | x |  |  |  | x |  |
| None | 5% | 0% |  |  |  |  |  |  |  |  |  |  |  |
| 1 to 2 | 10% | 40% |  |  |  |  |  |  |  |  |  |  |  |
| 2 to <10 | 75% | 60% |  |  |  |  |  |  |  |  |  |  |  |
| Up to 10 | 5% | 0% |  |  |  |  |  |  |  |  |  |  |  |
| Unknown | 5% | 0% |  |  |  |  |  |  |  |  |  |  |  |
| **Reasons for sleep disturbance** |  |  |  |  |  |  |  |  |  |  |  |  |  |
| Itching from AD | 35% | 30% |  |  |  | x | x |  |  |  |  |  |  |
| Itching from AD + other causes | 65% | 70% |  |  |  | x | x | x | x |  |  |  |  |

* These items do not assess a particular concept but are used to derive sleep metrics

Abbreviations: NWASO-AD = number of times of WASO not related to AD; SE = sleep efficiency; SOL = sleep onset latency; SQR = sleep quality/refresh; SSD = Subject Sleep Diary; TIB = time in bed; TST = total sleep time; TWT = total awake time; WASO = wakefulness after sleep onset; WASO-AD = duration of WASO related to AD
