# Supplementary material for: Capturing patient-reported sleep disturbance in atopic dermatitis clinical trials
Source: J Patient Rep Outcomes. 2024 Jul 15;8:73. doi: 10.1186/s41687-024-00751-7 (PMC11250737; doi:10.1186/s41687-024-00751-7)
Supplement: Supplementary file 3 — Supplementary Material 3 [file 41687_2024_751_MOESM3_ESM.docx]

Table S2. Concept mapping for evening SSD items: sleep disturbance concepts reported in the concept elicitation interviews and the SSD conceptual framework

|  |  |  | **SSS evening items (4 items)**  **No sleep metrics are derived based on the evening items** | | | |
| --- | --- | --- | --- | --- | --- | --- |
| **Results from Qualitative Interviews with Patients with AD** | **Adults**  **(N=20)** | **Adolescents**  **(N=10)** | **Q1e:**  **Number of naps during the day** | **Q2e:**  **During of naps during the day** | **Q3e:**  **Number of dozes during the day** | **Q4e:**  **During of dozes during the day** |
| **Description of sleep disturbance** |  |  |  |  |  |  |
| Daytime fatigue or sleepiness | 55% | 50% | x | x | x | x |
| **Impact on daily life** |  |  |  |  |  |  |
| Feeling tired, fatigued, or drowsy during the day | 95% | 50% | x | x | x | x |
| Impact on work or school | 30% | 60% | Not covered by SSD; can be assessed by another PRO (WPAI) | | | |
| Affects mood, increased irritability | 20% | 0% | Not covered by SSD; can be assessed by another PRO (HADS) | | | |

Abbreviations: WPAI: Work Productivity and Activity Impairment; HADS = Hospital Anxiety and Depression Scale
